# A model for dynamical switching in tristable perception for visual plaids

**DOI:** 10.1186/1471-2202-13-S1-P59

**Published:** 2012-07-16

**Authors:** Gemma Huguet, Jean-Michel Hupé, John Rinzel

**Affiliations:** 1 Courant Institute of Mathematical Sciences, New York University, New York, NY, 10012, USA; 2 CerCo, Toulouse University & CNRS, Toulouse, France; 3 Center for Neural Science, New York University, New York, NY, 10003, USA

## 

When observers view for extended time an ambiguous visual scene with two or more different interpretations they report switching between different perceptions. We focus on a classical paradigmatic stimulus, the visual plaids, consisting of two superimposed drifting gratings with transparent intersections [1,2]. For visual plaids, tristable perception is experienced: one coherent percept (the gratings move together as a single pattern) and two transparent percepts (the gratings slide across one another) with alternating depth order [3]. In order to decipher the complex mechanisms of tristable perception, we gathered a large amount of psychophysical data on tristable plaids and developed a neural network, firing rate model of interaction between neural populations that could account for the experimental results.

Nine subjects reported continuously the three possible percepts for 3 sessions of 10 stimuli each (3 minutes per stimulus). The angle between the vectors normal to gratings was equal to 80, 100 or 120 degrees. We collected enough percepts to compute statistics for each subject and parameter without collapsing data [4].

As opposed to bistable stimuli where the only possibility is the alternation between the two percepts, in the tristable case the results show that the next percept probability depends on the previous percepts. Indeed, the sequence of perceptual switches confirms that switches between two transparency states are typically interleaved by a coherent percept, especially for values of the angle equal to 80 and 100 [3,4]. Moreover, by examining triplets consisting of two transparent percepts interleaved by a coherent one, we observed that the probability of the two transparent percepts in the triplet having the same depth pattern decreases as the duration of the coherent percept shortens. These trends suggest that adaptation is implicated in perceptual alternations. For bistable alternations correlations are absent or insignificant.

We propose inhibition-based competition along with adaptation and noise in a multi-state framework as plausible mechanisms for the dynamics of perceptual switching. Our model is a firing rate model consisting of three mutually coupled populations of cells, each one encoding a different percept. It is based on the firing rate models for alternations during perceptual bistability [5]. We can explain the dependence in perceptual history by introducing an inhibition imbalance in the interactions between neural populations (the two transparent percepts inhibit each other more strongly than they inhibit the coherent state, making the latter more dominant and more likely to switch to). Adjusting the relative strength of adaptation and noise we can account for the dominance duration distributions and the switching probability between depth percepts as a function of the coherent percept duration. Finally, we consider other possible architectures for the model and we show that a non-hierarchical architecture where motion is encoded together with depth fits better with the experimental results.
